# Inflamm-Aging and Brain Insulin Resistance: New Insights and Role of Life-style Strategies on Cognitive and Social Determinants in Aging and Neurodegeneration

**DOI:** 10.3389/fnins.2020.618395

**Published:** 2021-01-14

**Authors:** Yulia Komleva, Anatoly Chernykh, Olga Lopatina, Yana Gorina, Irina Lokteva, Alla Salmina, Maik Gollasch

**Affiliations:** ^1^Charité - Universitätsmedizin Berlin, corporate member of Freie Universität Berlin, Humboldt-Universität zu Berlin, Berlin, Germany; ^2^Experimental and Clinical Research Center (ECRC), Charité - Universitätsmedizin Berlin, Berlin, Germany; ^3^Department of Biochemistry, Medical, Pharmaceutical & Toxicological Chemistry, Krasnoyarsk State Medical University named after Professor V.F. Voyno-Yasenetsky, Ministry of Health of the Russian Federation, Krasnoyarsk, Russia; ^4^Research Institute of Molecular Medicine and Pathobiochemistry, Krasnoyarsk State Medical University named after Professor V.F. Voyno-Yasenetsky, Ministry of Health of the Russian Federation, Krasnoyarsk, Russia; ^5^Medical Center “Private Practice”, Krasnoyarsk, Russia; ^6^Greifswald Medical School, University of Greifswald, Greifswald, Germany; ^7^Geriatric Medicine Center, Wolgast Hospital, Wolgast, Germany

**Keywords:** aging, inflammation, inflammasome, metaflammasome, insulin resistance, Alzheimer’s disease, anti-inflammatory strategies, calorie restriction

## Abstract

Over the past decades, the human life span has dramatically increased, and therefore, a steady increase in diseases associated with age (such as Alzheimer’s disease and Parkinson’s disease) is expected. In these neurodegenerative diseases, there is a cognitive decline and memory loss, which accompany increased systemic inflammation, the inflamm-aging, and the insulin resistance. Despite numerous studies of age-related pathologies, data on the contribution of brain insulin resistance and innate immunity components to aging are insufficient. Recently, much research has been focused on the consequences of nutrients and adiposity- and nutrient-related signals in brain aging and cognitive decline. Moreover, given the role of metainflammation in neurodegeneration, lifestyle interventions such as calorie restriction may be an effective way to break the vicious cycle of metainflammation and have a role in social behavior. The various effects of calorie restriction on metainflammation, insulin resistance, and neurodegeneration have been described. Less attention has been paid to the social determinants of aging and the possible mechanism by which calorie restriction might influence social behavior. The purpose of this review is to discuss current knowledge in the interdisciplinary field of geroscience—immunosenescence, inflamm-aging, and metainflammation—which makes a significant contribution to aging. A substantial part of the review is devoted to frontiers in the brain insulin resistance in relation to neuroinflammation. In addition, we summarize new data on potential mechanisms of calorie restriction that influence as a lifestyle intervention on the social brain. This knowledge can be used to initiate successful aging and slow the onset of neurodegenerative diseases.

## Introduction

It is a known fact that over the past decades, human life expectancy has greatly increased (Costantini et al., 2018). As a result, the population is aging, and this determines the development of geriatric medicine. Since aging is the main risk for the development of age-associated diseases, the field of geriatrics and geroscience has been developing very actively recently. The main goal of studies is to avoid age-related diseases before it is too late. Recently, the number of publications on anti-aging technologies and interventions has been increasing. This topic is certainly very popular not only in the medical community but also in society (Scapagnini et al., 2016).

Aging may be a complex process that happens under the influence of genetic, epigenetic, and environmental factors. Changes in an aging organism occur at the molecular, cellular, and tissue levels (Khan et al., 2017). In this regard, the question naturally arises on what factors possibly influence it. The most promising and effective approaches are nutritional strategies, physical activity, and hormone therapy (Scapagnini et al., 2016). In addition, these approaches can be used not only as anti-aging strategies but also as preventive directions. Preventive technologies will slow down aging and have a greater impact on quality of life than disease-specific approaches.

In order to understand the basis for development the directions of preventive and anti-age medicine, it is necessary to understand what basic pathological processes underlie the aging process. Some of these processes that determine aging include inflammation, cellular senescence, and senescence-associated secretory phenotype (SASP) development, altered glucose tolerance, and insulin resistance (IR) following dysregulated nutrient sensing and impaired cell–cell communication (De Souto Barreto et al., 2020). All these pathophysiological processes underlie age-associated neurodegenerative disorders.

It is predicted over the subsequent years that the incidence of age-related neurodegenerative diseases will increase dramatically. One of the most important factors in brain aging is the extremely high energy demand of neurons for maintaining neuronal work and preserving mental abilities (Davinelli et al., 2016).

With age, there is an increase in systemic inflammation, the inflamm-aging, and peripheral immunosenescence. Due to reciprocal interactions between the nervous and immune systems, chronic aseptic inflammation within central nervous system (CNS), called neuro-inflamm-aging, develops. Immunosenescence and inflamm-aging accompany brain aging and the loss of mental, cognitive, and other complex behaviors characteristic of Alzheimer’s disease (AD) and Parkinson’s disease (PD) (Khan et al., 2017; Costantini et al., 2018).

Recently, much research has been focused on the consequences of nutrients, and adiposity- and nutrient-related signals in brain aging and cognitive decline. Previously, it has been shown that insulin signaling affects the molecular cascades that underlie hippocampal functions, cognition, and memory (Spinelli et al., 2019). Our previous results have shown that a significant contribution to the development of brain IR is caused by neuroinflammation due to the overproduction of proinflammatory cytokines, astroglial and microglial activation, and disruption of the processes of reparative neurogenesis (Komleva et al., 2018).

The purpose of this review is to discuss current knowledge in interdisciplinary field of geroscience—immunosenescence, inflamm-aging, and metainflammation—which make a significant contribution to aging. A substantial part of the review is devoted to frontiers in the brain IR in relation to neuroinflammation. In addition, in this article, we summarize new data on potential mechanisms of calorie restriction (CR) influence as a lifestyle intervention on the social brain. This knowledge can be used to initiate successful aging and slow the onset of neurodegenerative diseases.

## Inflamm-Aging and Immunosenescence in Alzheimer’s Disease

### Inflamm-Aging and Immunosenescence

**FIGURE 1:**
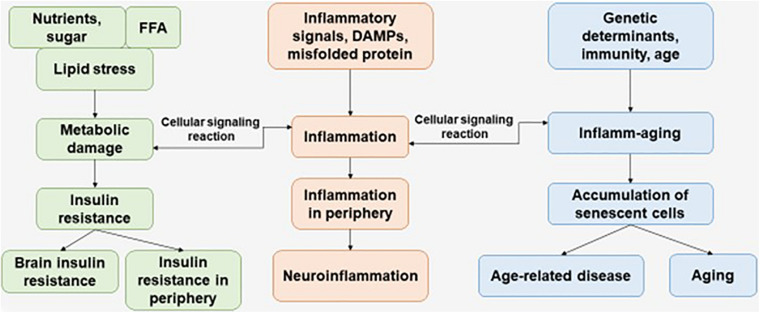
The interaction of factors initiating the development of metabolic damage, inflammation, and inflamm-aging. The aging process and age-related disease can be accompanied with central and peripheral insulin resistance, inflammation, and accumulation of senescent cells. FFAs, free fatty acids.

Immunosenescence is a phenomenon of irreversible loss of the ability to divide, as a result of which damage to the immune defense is observed, which contributes to the progression of susceptibility to disease in the elderly. Immunosenescence occurs when the ability to respond to new antigens is reduced (Pawelec, 2017). In addition, a variety of factors affect the progression of immunosenescence—genetics, environment, lifestyle, and nutrition—leading to infections and progression of disease pathology (Figure 1) (Costantini et al., 2018).

Franceschi et al. (2000) first used the term “inflamm-aging,” proposing a hypothesis based on a series of observations showing that aging of many organisms, including humans, is accompanied by an increase in the level of inflammatory markers in the blood, cells, and tissues. This is chronic, sterile, not associated with the presence of an infectious agent, and primarily due to endogenous signals, or subclinical (asymptomatic), mild, or basal inflammation, which is associated with aging. Already today, more and more studies indicate that “inflammatory” aging or inflammation associated with age is a risk factor for many chronic non-communicable diseases, such as cardiovascular (coronary heart disease and arterial hypertension), metabolic [diabetes mellitus 2 type (T2D)], musculoskeletal (osteoarthritis, osteoporosis, and sarcopenia), neurological (depression, dementia, and AD), and hematologic (malignant neoplasms and anemia) diseases, which leads to adverse effects on human health (Giunta et al., 2008). Many researchers agree that the number of nosologies is not limited to this list (Deleidi et al., 2015; Pawelec, 2017). The approaches for the treatment of diseases in which inflammation predominates in the pathogenesis may include, in addition to limiting caloric intake and increasing physical activity, the use of drugs. The drug action is aimed at limiting it by interfering with the processes of intracellular and extracellular signaling at different stages, and not only involves the impact on the main clinical manifestations or targets (blood pressure, cholesterol, blood sugar, etc.) (Zotkin et al., 2020).

### Alzheimer’s Disease and Neuroinflammation

AD is a fatal neurodegenerative disorder that is pathologically defined by extensive neuronal loss and the accumulation of intracellular neurofibrillary tangles (NFTs) and extracellular amyloid plaques in the brain. Studies since the discovery of amyloid beta (Aβ) and tau protein have provided detailed information on molecular pathological events, but little is known about the causes of AD, as well as about the possible effective treatment of this pathology (Silva et al., 2019; Tiwari et al., 2019).

It is well known that the risk of late-onset AD is partially due to genetics. In 2019, the results of a large meta-analysis of associations across the genome of clinically diagnosed late-onset AD were published. Currently, 25 risk loci are affected, five of which were identified in the latest study. Another confirmation of the contribution of the immune system to the late onset of AD confirms that the neurological and immune-mediated disease haplotype HLA-DR15 (human leukocyte antigen) is a risk factor for AD. In addition to tau-binding proteins and amyloid precursor protein (APP) metabolism, pathway analysis includes immunity and lipid metabolism, which are also associated with late onset of AD (Kunkle et al., 2019).

**FIGURE 2:**
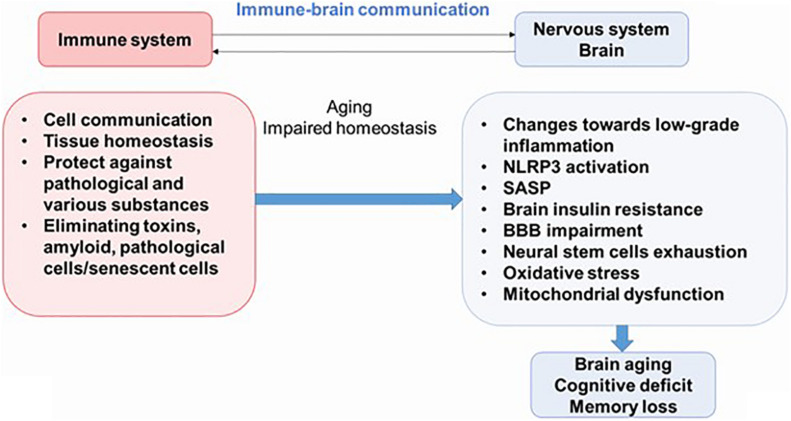
The immune–brain communication. The main functions of immune system are cell communications, tissue homeostasis, protecting against pathogens and various substances, and eliminating pathological cells. During aging, increased systemic inflammation leads to impaired homeostasis and could result to cognitive deficit. NLRP3, NLRP3 inflammasome; SASP, senescence-associated secretory phenotype; BBB, blood–brain barrier.

Newly discovered evidence proposes that inflammation is an important feature of AD, diabetes mellitus, and other pathologies; and it is believed that this process plays an important role in the pathogenesis of these syndromes. Therefore, understanding the interactions between the nervous system and the immune system may be key to preventing or delaying the onset of most diseases of the CNS. Neuroinflammation is an important part of the brain’s defense mechanisms against a variety of pathological agents, such as infections and injuries, and includes both the formation of soluble factors and the activation of specialized cells that are mobilized to respond and restore the brain’s normal physiology. Neuroinflammation is characterized by neuronal death in certain areas of the CNS (Figure 2) (Salmina et al., 2015; Calsolaro and Edison, 2016).

In AD, central events seem to combine the inflammasome, NF-κB pathway, and the microglial activation by a variety of factors, including Aβ and proinflammatory cytokines (Harms et al., 2015; Rea et al., 2018). Aβ in the brain microenvironment causes the glial activation that leads to microgliosis and astrocytosis around pathological proteins. Thus, glial cells are chronically activated in the brain before the onset of AD, which is associated with the development of chronic inflammation and contributes to the pathogenesis of AD. In the AD brain, microgliosis and astrocytosis because of the presence of senile plaques and NFTs can be detected immunohistochemically, and these glial cells exhibit pathologically specific morphology. Although the degree of gliosis correlates with cortical thickness and neurodegeneration, the role of various glial cells in neurodegenerative processes remains unknown (Saito and Saido, 2018).

Microglia, the main immune cells of the brain’s innate immunity, perform movements similar to macrophages to remove pathogens and protect neurons from various factors. At the same time, microglial cells secrete reactive oxygen species (ROS) and nitric oxide, which are neurotoxic. They also release proinflammatory cytokines and chemokines in response to danger signals. Dysregulation of microglial activity is associated with the pathogenesis of AD during aging (Salmina et al., 2015; Clayton et al., 2017). Triggering receptor expressed on myeloid cells-2 (TREM2) plays an important role in supporting microglial cell survival. Previously, it was shown that TREM2 promotes microglial clustering around fibrillar Aβ plaques in AD mouse model and postmortem human brain sections (Ulrich et al., 2017). Furthermore, TREM2 is a Aβ receptor that mediates microglial function and removal of Aβ (Zhao et al., 2018). An increase in soluble TREM2 fragments in cerebrospinal fluid indicates coincidence with markers of neuronal damage and onset of clinical dementia in AD (Ulrich et al., 2017).

**FIGURE 3:**
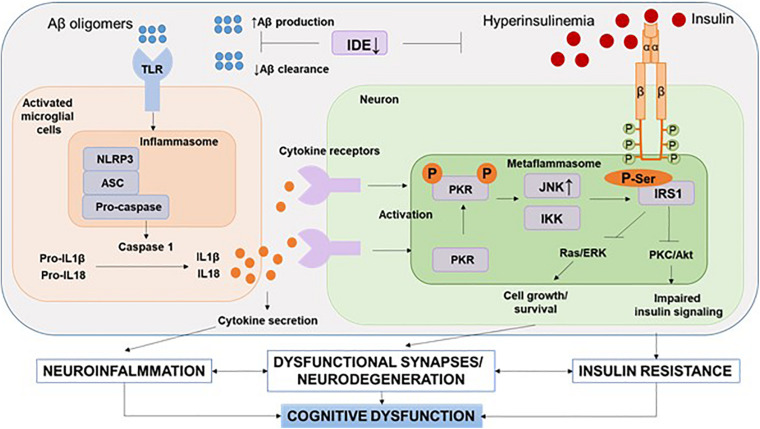
NLRP3 inflammasome and metaflammasome activation in response to DAMPs. In the brain, activation of toll-like receptors leads to the assembly of NLRP3 inflammasomes and the maturation and release of IL-1β. Through cytokine receptors, kinases are activated—components of metaflammasome—JNK and IKK. This leads to impaired insulin signaling and the development of insulin resistance, which leads to dysfunction of synapses and the development of neurodegeneration. Aβ, amyloid beta; Akt, protein kinase B; ASC, apoptosis-related speck-caspase recruitment domain; DAMPs, damage-associated molecular patterns; IDE, insulin degrading enzyme; IKK, IkBa kinase; IRS, insulin receptor substrate; JNK, c-Jun N-terminal kinases; NLRP3, NOD-like receptor pyrin domain-3 inflammasome; PKC, protein kinase C; PKR, double-stranded RNA-dependent protein kinase; Ras/ERK, kinase regulated by extracellular signals; TLR, toll-like receptors.

It should be noted that recent human positron emission tomography (Aβ-PET) data indicate that Aβ deposition begins years before memory impairment and cognitive decline (Hatashita and Wakebe, 2019). Given the fact that Aβ acts as strong damage-associated molecular patterns (DAMPs), it seems that the interval between early accumulation of Aβ and later signs of disease progression, such as tau pathology and brain atrophy, is influenced by innate immune responses (Table 1). One of the canonical pathways of this Aβ-induced innate immune response is the activation of the NOD-like receptor (NLR) family, a pyrin domain-containing 3 (NLRP3) inflammation, which has been the subject of intense research (Heneka et al., 2018) (Figure 3).

**TABLE 1:** Damage-associated molecular patterns (DAMPs), their receptors and molecular action in insulin resistance and Alzheimer’s disease.

DAMP	Receptors or sensors	Molecular action and effects	References
High mobility group box 1 (HMGB1) (alarmin)	RAGE, TLR4	Signal to the NF-κB signaling pathway and thus contributes to the inflammatory responses in type 2 diabetes mellitus, in the genesis and pathophysiology of IR and neurodegeneration.	Gonelevue et al., 2018; Paudel et al., 2020
Aβ (amyloid)	TLR4, TLR2, NLRP3, CD36, CD14 receptor	Aβ activates the NLRP1 and NLRP3 inflammasomes. The oligomers can disturb the functions of K^+^ channels, decreasing the intracellular K^+^ concentration and thus activating caspase-1. Increasing K^+^ efflux with valinomycin led to activated caspase-1 and IL-1β secretion from neurons. Aβ can also activate microglial cells in the brain through interaction with the surface receptor CD36, which induces the formation of a TLR2–TLR6 heterodimer and subsequently leads to NF-κB signaling.	Stewart et al., 2010; Heneka et al., 2018; Venegas and Heneka, 2019
Chromogranin A (CGA) (an acidic protein localized in secretory vesicles)	TLR4, CD14, or class A scavenger receptor	The stimulation of target receptors promotes the uptake of Aβ and phagolysosome formation. Upon lysosomal rupture, cathepsin B release is instrumental in the activation of procaspase-1 that ultimately produces IL-1β.	Lechner et al., 2004; Venegas and Heneka, 2019
ATP	P2 × 7R (an ATP-gated ion channel supporting Na^+^ and Ca^2+^ influx into and K^+^ efflux out of the cell)	The decrease in intracellular K^+^ leads to P2 × 7R-mediated NLRP3 inflammasome formation. Together with IL-1β release, NLRP3 inflammasome activation in the brain through the P2 × 7 receptor induces an increase of tau secretion in exosomes and its subsequent transmission to neurons.	Muñoz-Planillo et al., 2013; Asai et al., 2015; Venegas and Heneka, 2019
Ceramide (a sphingosine-based, lipid- signaling molecule that is formed from serine and 2 fatty acids)	NLRP3	Ceramide can act as an endogenous signal to caspase-1 cleavage and IL-1β secretion	Shin et al., 2015; Venegas and Heneka, 2019
S100	RAGE	Stimulate cell proliferation and migration and inhibit of apoptosis and differentiation, which participate in neurodegenerative processes. RAGE receptor activation leads to the activation the p38 MAPK cascade NF-κB.	Cristóvão and Gomes, 2019; Venegas and Heneka, 2019
mt-DNA and cf-DNA	TLR9 AIM2	Induce the release of interferon type 1 and TNF-α. Exogenous mtDNA fragments induced TLR9-mediated NF-κB activation in primary muscle cells. mtDNA increased TLR9 content in muscle cells. When cf-DNA binds to TLR, signaling occurs through MyD88, which leads to a type I IFN response. When cf-DNA binds to AIM2, caspase-1 is activated, and subsequently, IL-1β is released.	Shin et al., 2015; Venegas and Heneka, 2017; Yuzefovych et al., 2018
HSPs—heat shock proteins	PRRs (pattern recognition receptor). TLR2 and TLR4	Interaction with receptors leads to the induction of inflammatory cytokines such as TNF-α, IL-1β, IL-12, and GM-CSF.	Campanella et al., 2018; Venegas and Heneka, 2019
Homocysteine (Hcy)	NLRP3	Activation of the inflammasome with the subsequent release of interleukins. Hcy mediates the development of insulin resistance.	Smith et al., 2018; Zhang et al., 2018
Glucose	NLRP3	Induction of IL-1β secretion followed by increased apoptosis triggered by Fas via NF-κB and JNK and/or inhibiting insulin signaling.	Shin et al., 2015
IAPP [islet amyloid polypeptide (IAPP)–amylin]	NLRP3, CD36, and RAGE	IAPP has cytotoxic effects; assembly of the inflammasome leads to the formation of mature IL-1β.	Fawver et al., 2014; Shin et al., 2015
FFAs and their metabolites (palmitate)	NLRP3, TLR4	The production of inflammatory cytokines through activation of TLR and NLRP3 contributes to the development of insulin resistance by suppressing insulin signaling.	Shin et al., 2015

Studies have shown that Aβ oligomers can trigger the expression of the NLRP3 inflammasome and thus promote inflammation and intensify association between T2D and AD (Rea et al., 2018). Inflammasome is involved in the progression of metabolic syndrome due to impaired adipose tissue sensitivity. It has been conclusively demonstrated that obesity triggers NLRP3 activation and that secreted IL-1β that impairs insulin signaling, which contributes to IR in mice (Mori et al., 2011; Rea et al., 2018). Another study found that obesity was associated with NLRP3 activation in adipose tissue (Mori et al., 2011; Vandanmagsar et al., 2011; Rea et al., 2018).

The role of NLRP3 inflammasomes in the pathogenesis of obesity has been supported by data showing that Nlrp3^–/–^ and Asc^–/–^ knockout mice are protected from obesity and IR induced by a high-fat diet. In addition, NLRP3 activation by inflammasomes/caspase-1 appears to be a key regulator of adipocyte differentiation and directs adipocytes to an insulin-resistant phenotype (Stienstra et al., 2010).

Consequently, CR and weight loss in obese diabetic subjects decrease the expression of the Nlrp3 and IL-1 genes in adipocytes, improving insulin sensitivity (Vandanmagsar et al., 2011). However, some studies have failed to find an association between NLRP3 inflammasome formation and obesity or IR (Nishimoto et al., 2016). Understanding the molecular mechanisms of chronic inflammation remains a major medical problem (Nishimoto et al., 2016; Rheinheimer et al., 2017). Thus, further research is required to understand the relationship between NLRP3 inflammasome formation and IR.

Moreover, the inflammasome pathway is activated by a variety of intracellular processes and associated with increased age and age-related diseases. Both the inflammatory pathway and the senescent cell-related SASP activate the inflammasome through the NF-κB and IL-α cascade, causing the inflammatory response and cytokine production that delays resolution and healing (Chien et al., 2011; Mori et al., 2011; Rea et al., 2018).

Therefore, caspase-1 or inflammasome inhibitors have been proposed as novel treatments for pathologies associated with aging and metabolism deterioration (Stienstra et al., 2010; Kanbay et al., 2019).

## Metabolic Hallmarks of Aging and Their Role in the Cognitive Reserve

One of the most urgent tasks of modern gerontology is the search for various pathogenic factors that worsen the health and well-being of the elderly. Loss of function over time is distinctive for aging. Usually, the deterioration of the physical and mental condition occurs gradually. Incidentally, it is still not known whether this diminishment could be a result of physiological or pathological processes (Akintola and van Heemst, 2015).

Cognitive reserve is the determining factor in the difference between physiological and pathological brain aging. Cognitive reserve is related to the brain’s ability to maintain cognitive function despite being constantly under the influence of stressors and degenerative events associated with aging and the AD development. It is known that hippocampal neurogenesis is a lifelong process of continuous inclusion of functionally active new neurons into neuronal circuits. Accordingly, neurogenesis in the adult hippocampus is increasingly seen as a key factor in the sustainability of the cognitive reserve (Dainikova and Pizova, 2014). In addition, it was determined that the decisive factor in determining the resistance of nerve tissue to neurodegeneration is age-related decline in glial function and metabolic coupling. Thus, impairment of neuroglia and cell metabolism promotes the transition from physiological to pathological aging (Verkhratsky et al., 2015, 2020).

Risk factors that have been considered in relation to brain aging include metabolic disorders. The aging process of the brain can be accompanied with impaired glucose metabolism or decreased glucose supply to the brain. Moreover, brain IR has been associated with an increased risk of both cognitive decline and dementia, including AD and vascular dementia (Hughes and Craft, 2016; Yin et al., 2016; Baranowska-Bik and Bik, 2017) (Figure 1).

Currently, research has made significant progress in understanding the pathogenesis of AD but, unfortunately, without any disease-modifying therapeutics or proven prevention strategies. One of the most relevant and promising areas in terms of therapeutic effects is the study of brain metabolism. The leading opinion postulates that glucose metabolism is reduced in almost every neurological and psychiatric condition (Mosconi et al., 2009; Bélanger et al., 2011; Neth and Craft, 2017).

### Brain Insulin, Glucose, and Other Energy Sources for Thoughts During Aging

Until now, the production of insulin in the brain remains a controversial issue. There is a lot of conflicting evidence about the production of insulin in brain structures and cell types. The initial hypothesis was that insulin is able to cross the blood–brain barrier (BBB) through a saturable transport system. However, this mechanism is limited and ineffective. Later, data appeared on the possible production of insulin in the brain. Thus, the expression of mRNA and insulin protein was found in the hippocampus, olfactory bulb, striatum, hypothalamus, and entorhinal and prefrontal cortices (Mehran et al., 2012). Insulin secretion has also been reported in cultured astrocytes (Takano et al., 2018). In a recent study, the authors describe the presence of not only insulin mRNA but also the protein itself in the epithelial layer of the choroid plexus of mice and humans, along with proteins associated with the processing and secretion of insulin (Mazucanti et al., 2019).

Since the stability of the cognitive reserve is largely determined by neurogenesis, the study of metabolic trophic factors that influence this process is important. Insulin at moderate concentrations is known to play a neurotrophic role. Insulin has a pivotal role in the brain development, functioning of neurogenic niches, and aging. Activation of the insulin/IGF-I (insulin-like growth factor) signaling pathway regulates the exit of neuroblasts from the quiescence state. This signaling cascade, insulin and IGF-I, has been shown to promote neurogenesis by modulating proliferation, differentiation, and survival of neural stem cells (NSCs) (rev. in Spinelli et al., 2019). In healthy metabolic conditions, acute increases in insulin levels have a valuable effect on cognition. Nevertheless, chronically elevated insulin significantly reduces the level of its mediated effects (Neumann et al., 2008). Moreover, chronic hyperactivation of the insulin/IGF-I pathway can cause premature depletion of the stem cell pool (Spinelli et al., 2019). In contrast, high insulin levels may be associated with increased Aβ deposition in the brain, as insulin and Aβ compete for the same enzyme that provides their clearance, namely, the insulin-degrading enzyme (Hölscher, 2019). Thus, insulin can have a trophic or deleterious effect on neurogenesis (Spinelli et al., 2019). This conclusion can be confirmed by studies demonstrating impaired learning in animals with a model of type 2 diabetes mellitus (T2DM), as well as the observed cognitive deficit in clinical studies among patients with this pathology (Zilliox et al., 2016).

Recently, the term “type 3 diabetes mellitus” has often been used to denote AD, since the pathological events accompanying this pathology are pathogenetically associated with central IR (Kandimalla et al., 2017). Aβ suppresses insulin expression in astrocytes (Pitt et al., 2017; Spinelli et al., 2019). These data show bi-directional changes between impaired brain insulin signaling and Aβ deposition in AD. According to these results, local IR and changes in central glucose metabolism may be considered as early markers for the diagnosis of AD (Hölscher, 2019).

It was shown that central glucose hypometabolism can be detected decades before the clinical onset of AD (Mosconi et al., 2009; Sperling et al., 2011; Neth and Craft, 2017). However, in the last few years, it is becoming increasingly obvious as a condition of reactive or compensatory glucose hypermetabolism in neurologic diseases as an initial reaction to trauma and developing pathological processes (Ashraf et al., 2015; Neth and Craft, 2017). According to Neth and Craft (2017), a glucose hypermetabolism could be a temporary solution to the injury problem with a permanent reduction in glucose utilization. If additional data confirm the occurrence of an initial increase and a final decrease in glucose metabolism in the brain, then this shift can be visualized at an early stage (before the onset of clinical symptoms) and work to prevent the pathology. This hypermetabolic glucose shift at the early stages is likely complemented by increased use of other fuels as well. As the disease progresses, a bioenergy shift may occur due to a decrease in glucose dependence and an increase in the use of alternative energy sources (Neth and Craft, 2017).

As it has already been mentioned, the most preferred energy substrate for the brain, except the prolonged fasting, is glucose. For normal functioning of the brain, a constant supply of glucose is necessary. In small concentrations, glycogen was also found in the brain, which is stored in astrocytes. Glycogen provides lactate as an energy source for neurons through monocarboxylate transporters to support neural functions such as hippocampal-regulated memory formation and learning (Rich et al., 2019). Under conditions of reduced glucose intake, ketone bodies, mainly formed as a result of fatty acid (FA) oxidation, are an alternative main source of energy. In addition, FAs and their metabolites are capable of influencing many brain functions. This also allows them to be considered as potential targets for pharmacological and/or dietary interventions in certain brain pathologies (Romano et al., 2017).

Nevertheless, glucose is of the greatest importance as a brain energy substrate, and therefore, disturbances in glucose metabolism have significant consequences on the functioning of the brain. Decreased hippocampal volumes have been described in elderly with impaired glucose tolerance. It accompanied with a lower cognitive test performance. Similarly, another study confirmed that patients with higher fasting glucose and glycosylated hemoglobin experienced decreased memory and learning ability (Grabenhenrich, 2014). Glucose hypometabolism was most obviously recorded in the frontal, parietal, and temporal cortices (Tondo et al., 2020). This suggests that IR affects similar areas of the brain as in AD, supporting the notion that central IR may contribute to neurodegeneration. Moreover, diabetes has been repeatedly shown to be a strong predictor of cognitive dysfunction in the elderly (Kong et al., 2018).

Cognitive decline is not limited to impairments in learning and memory; there are other impairments as well. Brain IR has been confirmed to be associated with decreased task processing speed, cognitive flexibility, and motor skills. Current evidence confirmed that IR should be considered as an pivotal risk factor for the progression of cognitive dysfunction (Moheet et al., 2015).

Thus, clinical work and experimental studies in animals propose that IR has destructive effects on cognitive functions, in particular on learning and memory. Therefore, it becomes the principal aim to study the metabolic pathways and their association with the progression of AD and other neurodegenerative disorders (Spinelli et al., 2019).

### Brain Insulin Resistance in the Pathological Aging

Changes in brain insulin signaling, and in particular in the hippocampus, can alter molecular pathways involved in synaptic plasticity and neurogenesis in adults, thereby leading to a decrease in cognitive reserve, an increased risk of neurodegeneration, and a shortened life span (Epel, 2020). Long-term persistent excess of nutrients is the cause of stress acceleration of aging. However, an excess of nutrients causes hyperactivation of insulin signaling and leads to desensitization of IR-dependent molecular cascades. Because of this influence, the brain stops responding to insulin and eliminates both the metabolic and cognitive effects of this hormone (Spinelli et al., 2019).

IR makes it difficult for cells to maintain energy homeostasis. The brain in AD neurodegeneration is accompanied by changes similar to those observed in peripheral tissues in diabetes mellitus, including metabolic stress and neuroinflammation (Talbot et al., 2012). Thus, it can be assumed that such mechanisms explain IR in T2DM and impair central insulin transduction in patients with AD. Significant similarities between neuropathogenic mechanisms are induced by Aβ oligomers and cause the loss of neurons and synapses, as well as mechanisms associated with peripheral IR in diabetes (Craft, 2012).

Many explanations have been proposed for the impaired insulin transduction in the AD brain. One of them is decreased extracellular insulin assessed in cerebrospinal fluid, decreased total or cell surface of insulin receptor expression, and decreased affinity of insulin receptors for insulin (Talbot, 2014). The deficiency of extracellular insulin in the AD brain remains unclear and identified the opposite results obtained in cerebrospinal fluid (Molina et al., 2002). Similarly, deficits in total insulin receptors in AD brain tissues were not found in studies using age-matched controls, and cell fractionation did not reveal deficiencies in insulin receptor levels on the cell surface. Although the binding of insulin to the insulin receptor may be reduced in the brain tissue in AD, insulin still manages to activate the catalytic domain of the insulin receptor at a level of 71–74% of normal levels even in the hippocampal formation in AD. As noted above, a significantly greater decrease in insulin sensitivity is observed already after binding to the insulin receptor in the brain in AD. In the hippocampus, which is responsible for memory and learning, insulin activated only 10% of the normal level of insulin receptor substrate (IRS) (Talbot et al., 2012).

Thus, the most likely cause of decreased insulin signaling in the brain in AD is IR due to dysfunctional IRS-1. This appears to reflect Aβ-induced secretion of proinflammatory cytokines by glial cells. Among the early changes in AD, there is an increased solubility of Aβ, the monomers of which combine to form oligomers, which can later assemble into fibrils and form amyloid plaques (Sengupta et al., 2016). Also at the AD onset, Aβ oligomers activate microglia, which leads to the secretion of proinflammatory cytokines such as IL-1, IL-6, and tumor necrosis factor α (TNF-α) (Hemonnot et al., 2019). Such activation of microglia may play a key role in the AD pathogenesis, given the recent discovery that disabling a gene in AD model encoding a microglial receptor (i.e., NOD-like receptor 3) and perceiving inflammatory pathogens, including Aβ, prevent the development of AD and cognitive abnormalities that usually occur in this AD animal model (Heneka et al., 2013). Through neuronal receptors, microglial IL-1, IL-6, and TNF-α activate serine kinases IRS-1, known as IkBa kinase (IKK), C-Jun N-terminal kinase (JNK), and extracellular signal-regulated kinase (Erk2) (Talbot and Wang, 2014). Thus, Aβ oligomers injected into neuronal cultures or into the cerebral ventricles markedly increase the phosphorylation of IRS-1 serine (IRS-1 pS) at several sites, namely, S312, S616, and/or S636 (S307, S612, and S632 in rodents’ Erk2) (Talbot and Wang, 2014).

Elevated neural IRS-1 pSer is significant in the cortex and hippocampal formation in AD and, apparently, is the main cause of IRS-1 dysfunction in AD (Tanokashira et al., 2019). The most common cause of IR is inhibition of downstream signaling due to serine phosphorylation of the IRS1 (Boucher et al., 2014). Similar changes occur in the AD brain. Insulin activation of IRS-1 is consistently decreased in tissues with significantly increased levels of IRS-1 pS616 and IRS-1 pS636. These molecules may act as potential markers of central IR (Talbot et al., 2012; Talbot and Wang, 2014). As expected, the levels of these candidate biomarkers correlate significantly with the Aβ deposition and are associated with cognitive decline (Kong et al., 2018).

This may explain crossroad peripheral IR due to obesity and/or diabetes mellitus and brain IR in AD (Liu et al., 2011). Obesity and T2DM are, in fact, risk factors for AD and are associated with increased vascular proinflammatory cytokines (Pugazhenthi et al., 2017). With the development of AD, the impaired integrity of the BBB promotes the penetration of cytokines. They, in turn, activate IRS-1 serine kinases in the same way as cytokines obtained from microglia (Ferreira et al., 2018).

### Frontiers in Insulin Resistance Markers

The relationship between IR and AD may be enhanced due to a common etiology leading to an increased risk of AD (Spinelli et al., 2019). IR is a potentially modifiable risk factor for AD; in this regard, early diagnosis of IR remains highly relevant. Previous research has relied on measurements of systemic IR based on blood glucose and insulin values, such as the Homeostatic Model of Insulin Resistance (HOMA-IR) assessment (da Silva et al., 2019). Peripheral and central IR overlap to some extent, and this may explain why associations between HOMA-IR and glucose hypometabolism in the brain have been observed. Currently, a variety of proteins have been isolated that are consistently significantly related to IR and AD pathology. They are discoidin, CUB, and LCCL domain-containing protein 2 (DCBLD2); Ephrin-B2 (ENFB2); ciliary neurotrophic factor receptor subunit alpha (CNTFR); neuronal growth regulator 1 (NEGR1); leucine-rich repeat-containing protein 4B (LRRC4B); and SLIT and NTRK-like protein 4 (SLITRK4). However, a search for markers specific to central IR is also required (Westwood et al., 2017).

For these reasons, in recent years, research has focused on assessing glucose metabolism in the brain and analyzing extracellular brain vesicles extracted from blood as biomarkers of IR and early phase of cognitive decline. In a study in patients with IR, but without loss of cognitive functions, IR was associated with hypometabolism in the hippocampus and higher levels of blood pressure biomarkers in the cerebrospinal fluid (Westwood et al., 2017). Cerebral glucose metabolism is closely related to neuronal activity, and a decrease in the cerebral metabolic rate for glucose (CMRglc) is one of the main hallmarks of AD. *In vivo* imaging using 2-[^18^F]fluoro-2-deoxy-D-glucose PET (FDG-PET) demonstrates a consistent and progressive decrease in CMRglc in patients with AD. A decrease in CMRglc in preclinical stages of AD, with mild cognitive impairment (MCI) was also indicated, as well as in carriers of the allele of apolipoprotein E-epsilon-4, a strong genetic risk factor of AD (Mosconi et al., 2008).

In a recent study, Mullins et al. (2017) demonstrated that pSer312-IRS-1 (which stimulates uncoupling of IRS-1 and leads to its degradation) and p-panTyr-IRS-1 (which promotes insulin-stimulated responses) are biomarkers of AD. Based on these data, a methodology for the isolation of exosomes from plasma was developed, followed by immunoprecipitation against the cell surface adhesion protein L1-CAM to enrich neural origins. It was demonstrated that pathogenic and signal peptides in plasma exosomes expressing L1-CAM/NCAM effectively distinguish between AD and control group and can predict the diagnosis of the disease. In addition, it was confirmed that the peripheral IR is separated from the brain IR that occurs in AD (Mullins et al., 2017; Kapogiannis et al., 2019; Spinelli et al., 2019). These data indicate the presence of IR of the brain in patients with AD and, to a lesser extent, in people with diabetes (Mullins et al., 2017; Spinelli et al., 2019).

### Neuroinflammation and Brain Insulin Resistance

Inflammation is a feature of diabetes mellitus and AD, and it is believed that this process plays an important role in the pathogenesis of these two pathologies. Inflammation is an important part of the body’s defense mechanisms against many pathological agents, such as infections and trauma, and includes both the formation of soluble factors and the activation of specific cells that are mobilized to respond and maintain the normal physiology and homeostasis (Chatterjee and Mudher, 2018).

It is believed that similar inflammatory processes occur in the CNS and periphery. The presence of inflammatory markers in AD in the brain tissue, including increased levels of cytokines/chemokines, which accompany with gliosis, was confirmed in many studies (Cao, 2015). In addition, an increase of inflammatory mediators in the blood, such as TNF-α, IL-6, and IL-1β, was observed in patients suffering from AD (Ng et al., 2018). Moreover, increased production of proinflammatory cytokines in adipose tissue is a key feature of the pathogenesis of metabolic disorders. A recent study has shown that an elevated level of TNF-α expressed in adipose tissue of obese individuals is the reason of peripheral IR. Therefore, in both the central and peripheral tissues, uncontrolled or chronic inflammation accompanies IR (Saltiel and Olefsky, 2017). Interestingly, inflammation also underlies hypothalamic dysfunction in obesity (Samodien et al., 2019). New evidence indicates that it is the inflammation and stress of the endoplasmic reticulum that are critical pathogenetic events in the central and peripheral IR in metabolic disorders (Arruda et al., 2011). In an obese and diabetic animal model, the neuroinflammation, especially through activation of TNF-α and the IkBa kinase (IKK)-b/nuclear factor-kB pathway, is a principal mechanism underlying the disease pathogenesis (Milanski et al., 2009). Consequently, the pathology of the hippocampus in AD and the pathology of the hypothalamus in obesity have common pathogenetic pathways associated with inflammation (De Felice and Ferreira, 2014).

Our own data have shown the protective phenotype of Nlrp3 knockout mice in the development of brain IR (Chernykh et al., 2018). Using an experimental approach to modeling AD, we investigated new molecular mechanisms of insulin signaling dysregulation in the amygdala in association with neuroinflammation and emotional disorders. It has been established that experimental AD is accompanied by impaired expression and functional activity of molecules–components of insulin-mediated signaling pathways and the development of IR together with up-regulation of neuroinflammation in the brain amygdala. This cascade of pathological reactions is reflected in emotional behavior disorder. NLRP3-dependent mechanisms have been demonstrated in the basolateral amygdala in normal conditions and during the development of neurodegeneration. It has been experimentally proven that preventing the development of local IR by blocking NLRP3 inflammasomes should be considered an approach to correcting BIR and emotional disorders in AD. The studied molecular mechanisms linking the development of local IR with neuroinflammation (with the participation of pIRS, GLUT4, IRAP, and NLRP3) and impaired cognitive and emotional spheres open up new possibilities for the prevention and correction of neurodegeneration in AD.

Thus, suppression of neuroinflammation by preventing the expression of NLRP3 inflammasomes in *Nlrp3*-knockout mice has a protective role in the development of AD, accompanied by IR, due to modulation of the expression of pIRS-Ser and downstream of insulin signaling cascade (Gorina et al., 2019).

### Activation of Proinflammatory Factors and Signaling Pathways in a Cell Upon Damage to Insulin Signaling in Neurons in Alzheimer’s Disease

In peripheral IR, impaired TNF-α signaling results in JNK activation (Chen et al., 2015). Activation of the TNFa/JNK pathological pathway is associated with the main inflammatory and stress-signaling mechanisms, including tension of the endoplasmic reticulum and activation of stress kinases IKK (IkBa kinase) and PKR (double-stranded RNA-dependent protein kinase) (Nakamura et al., 2010). In T2DM, high levels of TNF-α initiate serine phosphorylation of IRS-1 by stress kinases, blocking insulin signaling (Nakamura et al., 2010). TNF-α levels increase in microvessels of the brain and cerebrospinal fluid in AD (Ruan et al., 2009). Initial information that impaired insulin signaling in neurons in AD is associated with proinflammatory signaling was based on the fact that oligomers of Aβ induce inhibition of IRS-1 through TNF-a/JNK activation (Bomfim et al., 2012). These ideas were confirmed, and it was shown that common mechanisms underlie damaged peripheral insulin transduction in diabetes mellitus and central local IR in the AD brain. Namely, it was shown that IKK and PKR were increased in AD in the brain, and they mediate the inhibition of IRS-1 in hippocampal neurons induced by oligomers of Aβ (Lourenco et al., 2013). IKK mediates neuronal inhibition of IRS-1 by Aβ oligomers (Bomfim et al., 2012).

## Metaflammation and Metaflammasome

As already described, assembly of the multiprotein complex inflammasome occurs not only in neurodegenerative diseases but also in metabolic disorders. The terms “metabolic inflammasomes” or “metaflammasomes” encompass metabolic disorders and the inflammation they cause. In other words, metaflammasome is a cascade of a signaling response in a cell caused by DAMPs followed by a metabolic pathway response and cytokine release (Table 1 and Figure 3) (Kanbay et al., 2019; Kuryłowicz and Koźniewski, 2020).

The expression of the four main components of the metaflammasome complex has been confirmed in the human brain. It includes phosphorylated forms of IKKβ, IRS1, JNK, and PKR (Taga et al., 2017).

C-Jun N-terminal kinases (JNKs) play an important role in a wide range of different stress-induced pathways. Thus, they are involved in neuronal cell death, migration, neuronal plasticity, autophagy, regeneration, metabolism, and regulation of cellular aging. Various stressors, including cytokines, ROS, growth factors, and Aβ oligomers, initiate the JNK signaling pathway. The role of JNK has been confirmed in studies of the relationship between neuronal death in AD and amyloid plaques. JNKs have been shown to increase Aβ production and are involved in the maturation and development of NFTs. In addition, it is currently considered a promising area of study of potential JNK inhibitors as a potential target for the treatment of neurodegenerative changes in AD (Yarza et al., 2016).

Studies have experimentally confirmed that low levels of JNK in T2DM are not accompanied by the development of cognitive impairments, including dementia. Conversely, a high level of C-Jun N-terminal kinases expression was recorded in patients with dementia and comorbid AD. In this regard, it has been convincingly demonstrated that JNK inhibition in the liver improves insulin signaling and reduces glucose tolerance. Kinase inhibition leads to a decrease in obesity and an increase in insulin sensitivity, while with the development of obesity, a pathological increase in expression is noted (rev. in Taga et al., 2017).

Another component of the so-called metaflammasome, which is involved in the regulation of inflammation, is IKKβ. IKKβ has a neuroprotective function by inhibiting neuronal NF-κB. This in turn protects neurons from Aβ and oxidative stress (Liu et al., 2017; Taga et al., 2017).

A recent study investigated the relationship of these four kinases. The data on the strength of the relationship between the components of the metaflammasome are compelling. Therefore, it was shown that there is no relationship between IKKβ and JNK kinases in the absence of dementia. At the same time, with the progression of AD, an inverse relationship is observed between IKKβ and JNK. There is no connection between IKKβ and IRS1 and PKR. This shows the special role of the relationship between the components of the metaflammasome, depending on the state, the presence of pathology, and the brain environment (Taga et al., 2017).

Another of the kinases of the so-called metaflammasome complex is a proapoptotic enzyme—eukaryotic initiation factor 2α kinase 2 (PKR). PKR inhibits translation and participates in cellular signaling, which leads to brain damage in AD and impaired memory consolidation. Aβ results in the activation of PKR and its accumulation in degenerating neurons. PKR modulates Aβ synthesis through the induction of beta-site APP-cleaving enzyme 1 (BACE 1). An elevated level of PKR was observed in the cerebrospinal fluid in AD patients and patients with an MCI. PKR activation leads to downstream cascade resulting in TNFα and IL1-β production. It was also shown experimentally that PKR inhibits molecular processes of memory consolidation. This kinase is also currently considered as a potential target for inhibition, can reduce neuronal death, and can facilitate cognitive decline in neurodegeneration (Hugon et al., 2017).

It was reported by Taga et al. (2017) that high expression of IRS1 and PKR is associated with cognitive impairment but not dementia. There are suggestions that the components of the metaflammasome can be activated precisely in the early stages of AD or in MCIs, which also makes these kinases promising for use as marker molecules for early diagnosis of degenerative events. This is confirmed by experimental animal studies, where after the intervention of a high-fat diet with the development of IR, an increase in IRS1 in the mice hippocampus was noted, which was accompanied by a deficit in spatial working memory due to postsynaptic impairment (Arnold et al., 2014; Spinelli et al., 2019).

In general, the metaflammasome hypothesis is based on the concept that dysfunction of the endoplasmic reticulum (due to the accumulation of unfolded proteins) leads to the expanded protein reaction and increased inflammation (Taga et al., 2017).

In addition, immunometabolic pathways are sensitive to lipids and are associated with lipotoxicity, which in turn causes metaflammation and changes in lipid metabolism (Ertunc and Hotamisligil, 2016). Since insulin is an important regulator of lipid metabolism as well, dyslipidemia is one of the main features of IR. The characteristic of peripheral IR is an increased content of free FAs (FFAs), an increase in the level of very-low-density lipoprotein (VLDL), and a decrease in high-density lipoprotein (HDL) (Kamagate et al., 2008; Neth and Craft, 2017). The dyslipidemia plays a role in amyloid deposition in AD, probably due to the effect of cholesterol on Aβ processing in the brain (Berti et al., 2015; Neth and Craft, 2017). This was confirmed by PET imaging. In addition, various genetic studies have identified several genes involved in lipid and cholesterol metabolism as increasing the risk of AD. This is primarily apolipoprotein-E (ApoE), followed by apolipoprotein-J (APOJ or clusterin, CLU), ATP-binding cassette subfamily A member 7 (ABCA7), and sortilin-like receptor. These results suggest a potential link between dyslipidemia and the accumulation of cerebral amyloid, which, in turn, may be mediated by IR, as well as other causes of lipid metabolism disorders, such as carriage of the Apoe4 allele (Neth and Craft, 2017).

ApoE is the main apolipoprotein, produced in the CNS, and directly increases the risk, progression, and pathogenesis of AD. Allele differences in ApoE confer specific effects on Aβ deposition, degradation and clearance, tau phosphorylation, neuronal damage, and inflammation (Stukas et al., 2015). There is evidence that the carriage of the ApoE4 allele can contribute to a decrease in insulin signaling by directly interacting with insulin receptors, leading to the uptake of insulin receptors within endosomes (Zhao et al., 2017). The role of clusterin (apolipoprotein J) in the risk and severity of AD was confirmed in relation to both cognitive function and Aβ metabolism. ApoA-I can also influence the pathology of AD, potentially by modulating cerebrovascular integrity and function, aiding in the removal of Aβ peptides from the cerebrovascular smooth muscle cells and reducing inflammation (Stukas et al., 2015).

### Anti-inflammatory Strategies Targeting Neurodegeneration and Metaflammation

Since the important role of inflammation in the pathogenesis of neurodegeneration and IR in various pathologies, including diabetes mellitus and AD, has been convincingly shown, approaches based on an anti-inflammatory strategy can be used to treat symptoms and to interrupt the vicious circle of metaflammation. As visceral fat is strongly linked to metabolic disorders, strategies for correcting IR in metabolic-cognitive states are very promising approaches as well (Kullmann et al., 2020).

There is epidemiological evidence that some anti-inflammatory approaches, in particular the use of non-steroidal anti-inflammatory drugs (NSAIDs), reduce the risk of AD. However, this category of drugs does not affect cognitive function (Rivers-Auty et al., 2020). In clinical trials, no evidence of the effectiveness of NSAIDs was found. The ineffectiveness of anti-inflammatory strategies may be due to inappropriate non-steroidal anti-inflammatory drugs or due to epidemiological results caused by confounding factors. However, there is evidence that, for example, the use of diclofenac is associated with a decrease in morbidity, as well as with a slower decline in cognitive function. However, this requires further research into the potential therapeutic effects of diclofenac in AD. Some antidiabetic drugs, which are aimed at lowering blood sugar levels, also have anti-inflammatory effects. This action is also associated with the hypolipidemic effect and direct modulation of immune responses. Despite promising results from clinical trials of anti-inflammatory drugs, salicylates, no clear guidelines have been established regarding the recommendation of these compounds for the prevention or treatment of T2DM. The use of other NSAIDs to combat metaflammation also requires clinical trials. However, most of the known methods of treating T2DM exhibit anti-inflammatory properties to varying degrees, which arise because of triggering various pathways and their effects, depending on many factors. Therefore, further clinical studies are needed to test new drugs and identify specific molecular pathways that could be therapeutically targeting metainflammation (Kuryłowicz and Koźniewski, 2020; Rivers-Auty et al., 2020).

There is evidence of a decrease in systemic inflammation with the applying of dietary protocols in clinical trials, associated primarily with a reduction in calorie intake (Lopez-Garcia et al., 2004; Kuryłowicz and Koźniewski, 2020). High-fat diets and high calories cause metainflammation, so the idea that dietary intervention can help reduce inflammatory the response in IR is very promising. Although the studies were not uniform in design, all reported that weight loss, improved glycemic control, and hepatic steatosis were associated with varying degrees with serum C-reactive protein (CRP) reduction (Kuryłowicz and Koźniewski, 2020).

Since it is a well-known fact that excess of nutrients in the course of obesity and IR impairs metabolism leading to the endoplasmic reticulum stress, possible CR may have a protective role (Park et al., 2012; Kuryłowicz and Koźniewski, 2020; Ma et al., 2020).

Calorie restriction is one of the most promising approaches for reducing the negative effects of metabolic disorders, age-related diseases, and pathologies associated with metaflammation (Park et al., 2012; Ma et al., 2020). In animal studies, it has been shown that reducing calorie intake increases life span and helps to lower blood glucose and insulin levels (Redman and Ravussin, 2011; Kim et al., 2020). There is currently evidence that CR for 2 years by 15% in healthy, non-obese people leads to a decrease in systemic oxidative damage (Redman et al., 2018). The main effects of CR in mammals include weight loss, improved insulin signaling by increasing hormone receptor sensitivity, normal lipid profiles, and increased adiponectin levels (Balasubramanian et al., 2017).

Thus, research data demonstrate the beneficial effects of CR in conditions such as diabetes, inflammation, obesity, and cardiovascular disease (Redman et al., 2018). However, the mechanisms underlying such changes remain unclear. An increase in adipose tissue is associated with the development of age-related metabolic changes, including the development of IR. In contrast, a decrease in adipose tissue during prolonged CR led to an improvement in age-related IR (Escrivá et al., 2007; Fabbiano et al., 2016; Corrales et al., 2019). CR slows down and restores age-related immunosenescence by regulating energy metabolism and oxidative stress and decreasing the production of proinflammatory cytokines and neuroendocrine homeostasis (Costantini et al., 2018).

## Calorie Restriction and Cognitive and Social Determinants of Aging: CD38 Signaling Mechanism in Aging and Neurodegeneration

**FIGURE 4:**
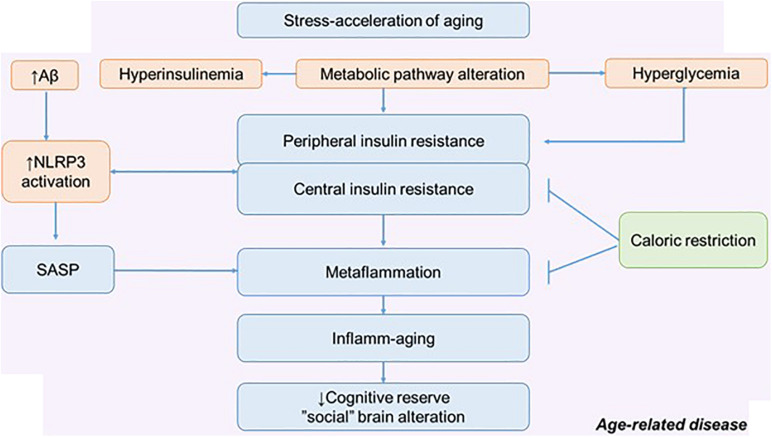
The metabolic pathway alteration in the development of metaflammation and inflamm-aging. Possible role of calorie restriction in preserving cognitive reserve and social behavior. NLRP3, NLRP3 inflammasome; SASP, senescence-associated secretory phenotype.

Inflammation, metainflammation, central and peripheral IR are determinants of aging along with behavioral, social, environmental, toxic, and other factors (Vidaček et al., 2018; Lever-van Milligen et al., 2019; Epel, 2020) (Figure 4). Different types of stressors can potentially lead to adaptive changes or accelerated aging. It depends on the nature of the stressors, stress resilience, and the stress response. At the same time, one of the promising strategies of slowing aging is an increase in stress resistance due to boosting stress resilience (Epel, 2020).

In relation to age and aging (pathological and physiological), many intervention approaches are considered. There are many potential approaches that may improve stress resilience: lifestyle interventions and CR are thought to work in part through adaptive response. Excess nutrients, high-fat diets, excessive calorie intake, and the traditional American diet can act as a stress acceleration of aging and lead to defective behavioral health. Therefore, CR can be considered as a stress rejuvenescence. The various effects of CR on metainflammation, IR, and neurodegeneration have been described (Hotamisligil, 2017; Epel, 2020). Less attention has been paid to the social determinants of aging and the possible mechanism by which CR might influence social behavior (Pifferi et al., 2018).

The positive effect of CR on cognitive longevity has been described, including through the effect on the morphological and functional properties of astroglia. Experiments on mice have shown that CR increases astroglial complexity and improves synaptic plasticity. Accordingly, this approach can increase neural compensation and cognitive reserve contributing to the healthy aging (Figure 4) (Verkhratsky et al., 2020).

One possible mechanism for mediated neuroprotection of CR is by regulating Ng-associated Ca^2+^ signaling. It causes a decrease in CaMKII and calpain activity, as well as downstream signaling that regulates neuronal metabolism, survival, and plasticity (Kim et al., 2016). CR significantly enhances cerebral blood flow and BBB function in young mice by reducing rapamycin expression, enhancing endothelial nitric oxide synthase signaling, and increasing ketone body utilization. This promotes memory formation and learning ability during aging and reduces anxiety in aging mice (Parikh et al., 2016). In a recent published study, the effect of CR on the social brain function was determined. In the long-term study CALERIE 1, CR did not change mood, but CR enhanced mood in patients in CALERIE 2, as well as improvements in tension anxiety were detected (Dorling et al., 2020). At the same time, there is also evidence that CR accelerated gray matter atrophy in old mouse lemurs but protected old animals from white matter atrophy in comparison with old control animals (Pifferi et al., 2018).

Currently, the mechanism of the CR influence on social behavior remains relevant and unexplored. A potential mechanism of this action may be the restoration of NAD^+^ through the activation of sirtuins and changes in the expression of NADase-CD38 (Tarragó et al., 2018). It was revealed that CD38 could act as a potential pharmacological target to reverse age-related NAD^+^ decline. NAD^+^ is an energy metabolism booster. CR affects AMP-activated protein kinase (AMPK) activity, which can modulate the bioavailability of NAD^+^ (Connell et al., 2019).

Calorie restriction activates sirtuins, suppresses signaling of growth hormone/insulin-like growth factors and mTORC1 (mammalian target of rapamycin), and enhances mitochondrial redox regulation (Zullo et al., 2018). Sirtuins are a family of proteins with NAD^+^-dependent enzymatic activities. Sirtuins regulate various cellular processes including glucose production, insulin sensitivity, inflammation, DNA repair, fat differentiation, FA oxidation, neurogenesis, and aging (Lee et al., 2019).

Previous research has reported a link between sirtuins and mitochondrial function and abnormal tau proteins and amyloid. It was confirmed that SIRT1, 3, and 6 are involved in age-related disease and regulation of life span, as well as AD progression (Hoshino et al., 2018). In mice model of AD, accompanied by impaired DNA repair, the precursor of NAD^+^, nicotinamide riboside (NR), increases SIRT3 and SIRT6 (Hou et al., 2018). The role of sirtuins has been proven not only in the development of inflammation but also in IR. SIRT1 activation leads to the suppression of metaflammasome components, namely, JNK and IKK (Yoshizaki et al., 2010).

As already noted, neurons have a high-energy demand, and therefore, they are very sensitive to a decrease in NAD^+^ and disruption of ATP production. NAD^+^, by increasing the sirtuins, affects neuronal survival, contributing to the maintenance of a balance between mitochondrial biogenesis and mitophagy (Kerr et al., 2017). These arguments are also supported by the fact that selective overexpression of SIRT1 and SIRT6 in transgenic mice increases the life span of animals. The application of NAD^+^ precursors [NR, nicotinamide mononucleotide (NMN), and nicotinamide] has a positive effect in neurodegenerative diseases and aging. The restoration of the NAD^+^ level led to an increase in life expectancy in different animal species (Zhang et al., 2016; Hou et al., 2018). In this regard, it is logical to assume that the restoration of NAD^+^ can act as a promising approach to the treatment of AD by influencing the pathology of tau protein and inflammation, as well as DNA repair (Hou et al., 2018).

Moreover, it is considered that NAD^+^ depletion not only is characteristic of AD but also occurs during physiological aging; the NAD^+^ precursor suppliers to medical nutrition leads to an improvement in cognitive functions and synaptic plasticity in AD (Gong et al., 2013). It is known that the enzymes involved in the degradation of cellular NAD^+^ are CD38 and PARP. It is believed that CD38 is the main NADase in mammalian tissues; in addition, CD38 and its homolog BST-1/CD157 degrade both NMN and NR. Thus, CD38 and BST-1 are involved in the regulation of cell metabolism, regulation of sirtuin activity, and signaling. It should be noted that genetic or pharmacological ablation of CD38 protects against metabolic dysfunction caused by a diet high in fat and calories by modulating SIRT1 activity (Chini et al., 2017). The studies in animals suggest that increasing tissue NAD^+^ levels by genetic CD38 ablation can significantly alter energy homeostasis in animals fed a calorie-excessive diet (Chiang et al., 2015).

At the same time, the physiological functions of CD38 in the brain have also been established. Thus, CD38 has been shown to play an important role in the secretion of oxytocin (OT) in the hypothalamus and in the regulation of social memory and social interactions (Higashida et al., 2019). In addition, recently, our group, together with Japanese colleagues, showed that NR corrects social deficits, as well as anxious behavior in CD157 knockout mice. These results suggest that increasing NAD^+^ levels with NR may allow animals with a deficiency of cyclic ADP-ribose and oxytocin to overcome this deficiency and function normally (Gerasimenko et al., 2020). This is supported by evidence that peripheral OXT administration improves social recognition, object recognition, and depressive behavior in high-fat-treated mice (Hayashi et al., 2020).

Therefore, diet-based strategies toward to CRs could be a promising therapeutic approach against AD, by influencing many signaling pathways, resulting in increasing cognitive reserve and maintaining social function (Esposito et al., 2015; Alkhatib et al., 2017; Wakabayashi et al., 2019; Kuryłowicz and Koźniewski, 2020).

## Conclusion

Thus, inflamm-aging is one of the manifestations immune aging and is a risk factor of morbidity and mortality among the elderly people. Since various chronic diseases associated with age are directly associated with inflammatory response, the approaches to decrease metaflammation could considered as intervention in age-related pathology. Despite numerous studies in age-associated pathologies, studies of the contribution of the components of congenital immunity in healthy aging are insufficient. It remains unclear whether the inflammatory phenotype is a manifestation of healthy aging or is associated with development age-related pathology. Moreover, given the role of metainflammation in neurodegeneration, lifestyle interventions such as CR may be an effective way to break the vicious cycle of metainflammation and have a role in social behavior.

## Author Contributions

YK: conception and design. YK and AS: literature review and drafting the article. AC, OL, YG, and IL: critical revision for relevant intellectual content. YK, AS, and MG: final approval of the version to be published. All authors have read and agreed to the published version of the manuscript.

## Conflict of Interest

The authors declare that the research was conducted in the absence of any commercial or financial relationships that could be construed as a potential conflict of interest.
